# Early detection of cryptic memory and glucose uptake deficits in pre-pathological APP mice

**DOI:** 10.1038/ncomms11761

**Published:** 2016-06-01

**Authors:** V. Beglopoulos, J. Tulloch, A. D. Roe, S. Daumas, L. Ferrington, R. Watson, Z. Fan, B. T. Hyman, P. A. T. Kelly, F. Bard, R. G. M. Morris

**Affiliations:** 1 Centre for Cognitive and Neural Systems, Edinburgh Neuroscience, The University of Edinburgh, 1 George Square, Edinburgh EH8 9JZ, UK; 2 Department of Neurology, Mass General Institute for Neurodegeneration, Massachusetts General Hospital, Harvard Medical School, 114 16th Street, Charlestown, Massachusetts 02129, USA; 3 Neuroscience Paris Seine, Institut de Biologie Paris Seine, Sorbonne Universités, Pierre and Marie Curie University, UM CR18, INSERM U1130, CNRS UMR 8246, 75005 Paris, France; 4 Department of Dietetics, Nutrition and Biological Sciences, School of Health Sciences, Queen Margaret University, Musselburgh EH21 6UU, UK; 5 Janssen Prevention Center, Janssen Pharmaceutical Companies of Johnson & Johnson, 3210 Merryfield Row, San Diego, California 92121, USA; 6 Instituto de Neurociencias, CSIC-UMH, San Juan de Alicante 03550, Spain

## Abstract

Earlier diagnosis and treatment of Alzheimer's disease would greatly benefit from the identification of biomarkers at the prodromal stage. Using a prominent animal model of aspects of the disease, we here show using clinically relevant methodologies that very young, pre-pathological PDAPP mice, which overexpress mutant human amyloid precursor protein in the brain, exhibit two cryptic deficits that are normally undetected using standard methods of assessment. Despite learning a spatial memory task normally and displaying normal brain glucose uptake, they display faster forgetting after a long delay following performance to a criterion, together with a strong impairment of brain glucose uptake at the time of attempted memory retrieval. Preliminary observations suggest that these deficits, likely caused by an impairment in systems consolidation, could be rescued by immunotherapy with an anti-β-amyloid antibody. Our data suggest a biomarker strategy for the early detection of β-amyloid-related abnormalities.

## Abstract (web summary)

Identifying early signs of Alzheimer's disease is important when it comes to diagnosis and treatment. Here, the authors identify subtle memory retrieval deficits and associated brain glucose uptake impairments in very young mouse models of Alzheimer's, prior to plaque development.

## Graphical abstract

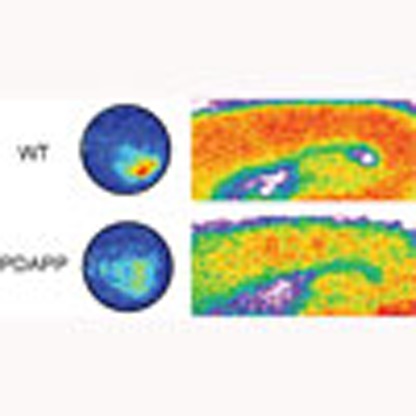

Identifying the onset of Alzheimer's disease (AD) as early as possible is now widely recognized as a crucial step in the development of effective treatments for the disease. It is faced, however, with the obstacle that the incipient cognitive, physiological and biochemical deficits are extremely difficult to observe^1^,^2^,^3^,^4^,^5^,^6^. Several biomarker approaches are available including biochemical analysis of cerebrospinal fluid or peripheral blood, imaging of Aβ plaques in the brain, magnetic resonance imaging and glucose uptake monitoring using positron emission tomography^7^,^8^. These have been very useful in patient studies, but prognostic diagnosis will likely require higher sensitivity and/or convergence towards multiple biomarkers^3^,^9^,^10^,^11^.

Studies with animal models derived from the genetics of familial AD^12^,^13^,^14^,^15^,^16^ suggest that subtle changes occur in living neurons, their synapses and networks, at a stage well before cell death^5^,^6^,^17^,^18^,^19^,^20^. Despite understandable scepticism about such models, these findings provide important insights towards pre-pathological biomarkers for AD that could include subtle alterations in behaviour and/or cerebrovascular metabolism. Spatial learning and memory in animal models have relevant validity as deficits in this aspect of behaviour are among the earliest symptoms observed by patients' families^21^. Mediated by the hippocampus and entorhinal cortex in rodents^22^,^23^, spatial learning requires effective hippocampal synaptic plasticity at the time of initial memory encoding^24^. However, lasting changes in synaptic structure that are the basis of memory consolidation and storage engages the neocortex^25^,^26^, including regions that are at risk in the earliest stages of AD. Memory encoding may be normal in the prodromal phase of AD, despite the ability for lasting changes in synaptic structure and function already being at risk^27^,^28^. A simple if indirect biomarker of synaptic efficacy is glucose uptake imaging. We wondered if this could be used in association with behavioural protocols that distinguish initial encoding from later stages of consolidation in a brain-wide manner.

The core idea of this study is that it might be possible to identify cryptic changes in spatial memory in a rodent model at a very young age when memory encoding is apparently normal. This would, in humans, correspond to a time when the disease process is at a pre-diagnosed stage. Using a familial AD genetic model (the PDAPP mouse), we trained very young (3–4 months) transgenic and littermate wild-type (WT) control mice in a watermaze^29^ using a spatial learning protocol explicitly designed to dissect learning from forgetting^30^,^31^,^32^. This involved training each mouse to a pre-determined criterion of reliable rapid escape from the water irrespective of how many training trials this might take (Fig. 1a). Memory retrieval could then be tested immediately, or consolidation allowed to proceed, with retrieval tested after a long delay. This behavioural protocol, in which some animals were allowed to complete only part of the stages, was combined with analysis of glucose uptake in the brain timed to be specific to distinct phases of memory processing. We also conducted preliminary experiments to confirm the specificity of our observed cognitive and physiological phenotypes to Aβ by a rescue experiment using immunotherapy.

## Results

### Normal learning and glucose uptake in young PDAPP mice

**Figure 1:**
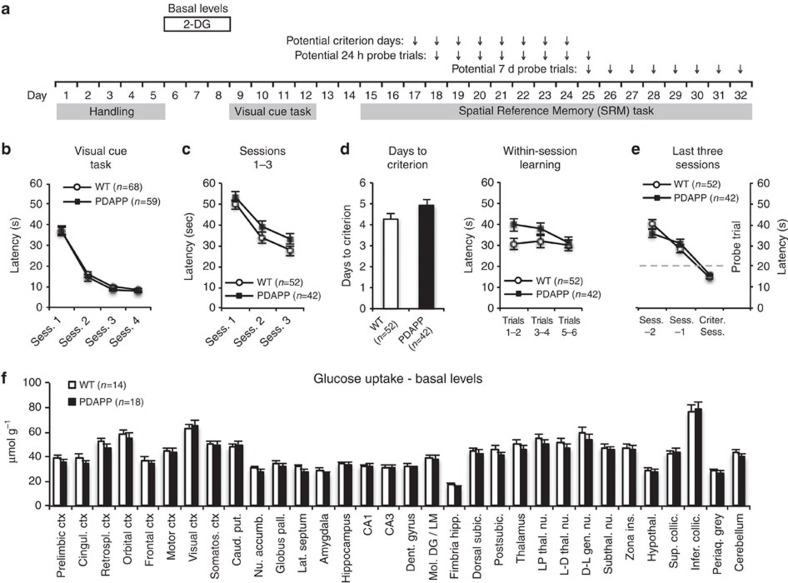
Normal learning and basal glucose uptake in young PDAPP mice. (**a**) Experimental design and training stage time points outlining our use of the training-to-criterion protocol. (**b**) PDAPP and WT mice (total *n*=127) learned to perform the visual cue task equally well (not significant). (**c**) Learning curves for sessions (Sess.) 1–3 of SRM training (days 15–17 in a) showed no significant difference between PDAPP and WT mice (ANOVA, between subjects comparison, F_1,92_=2.78, *P*=0.10). (**d**) There was a small but significant difference in the number of Sess. of SRM training to reach criterion (*t*_92_=1.84, *P*=0.035), associated with a trend suggesting overnight forgetting in PDAPP mice as shown in the within-Sess. escape latencies across Sess. 2 and 3 (groups × blocks: F_2,184_=2.98, *P*=0.054). (**e**) The PDAPP mice did not differ in escape latency as they approached criterion (Sess. −2 to the Criterion Session (Criter. Sess.): between-groups comparison, F_1,92_<1). (**f**) Basal glucose uptake (with no watermaze procedures) in 32 brain structures of young PDAPP and WT mice. No significant group differences were identified: between-subjects comparison, F<1; genotype × brain-region, F_3.4/100.5_=1.40, *P*>0.10 Greenhouse-Geisser correction (Student's *t*-tests: *P*>0.05 for all individual brain structures). Means±1 s.e.m. Accumb., accumbens; Caud., caudate; Ctx, cortex; Dent., dentate; gen., geniculate; hipp., hippocampus; Hypothal., hypothalamus; ins., inserta; Lat., lateral; Mol. DG/LM, molecular dentate gyrus/lacunosum moleculare; Nu., nucleus; pall., pallidus; Periaq., periaquedactal; Postsubic., postsubiculum; Prel., prelimbic; put., putamen; Retrospl., retrosplenial; Somatos., somatosensory; subic., subiculum; Subthal., subthalamic; Sup. collic., superior colliculus; thal., thalamic.

We trained a total of 127 mice (3–4 months; PDAPP *n*=59; WT *n*=68) through a varying number of stages of training (Fig. 1a). Training to a visible escape platform in the watermaze was normal (Fig. 1b), as was swim speed (Supplementary Fig. 1). Spatial learning to a hidden escape platform proceeded effectively over days 1–3 of training (from day 4 onwards the mice that reached the criterion of all daily trials averaging <20 s were not trained further; Fig. 1c). There was a small but significant difference in the number of days to reach criterion, apparently due to overnight forgetting that resulted in higher escape latencies by PDAPP mice on daily trials 1–2 (Fig. 1d), suggesting that learning *per se* is largely normal. In addition, the two groups showed equivalent navigational performance as they approached criterion (Fig. 1e and see Supplementary Fig. 2 for details) as revealed by a ‘backward' learning graph^33^. Thus, by analogy to presymptomatic AD patients, young PDAPP mice harbouring low levels of soluble Aβ (see below) can learn a hippocampal-dependent task effectively.

Basal glucose uptake in separate animals injected with [^14^C]-2-deoxyglucose (2-DG)^34^, measured across 32 brain areas (example image in Supplementary Fig. 3), indicated no difference across groups (Fig. 1f). This is in contrast to deficits previously observed in older PDAPP mice at a pathological age^35^. Thus, in addition to being normal in their capacity to learn, there are no major changes in brain glucose metabolism in these pre-pathological PDAPP mice. Histochemical analysis also confirmed the absence of Aβ plaques at this young age (Supplementary Fig. 4).

### Impaired memory retrieval and associated glucose uptake

**Figure 2:**
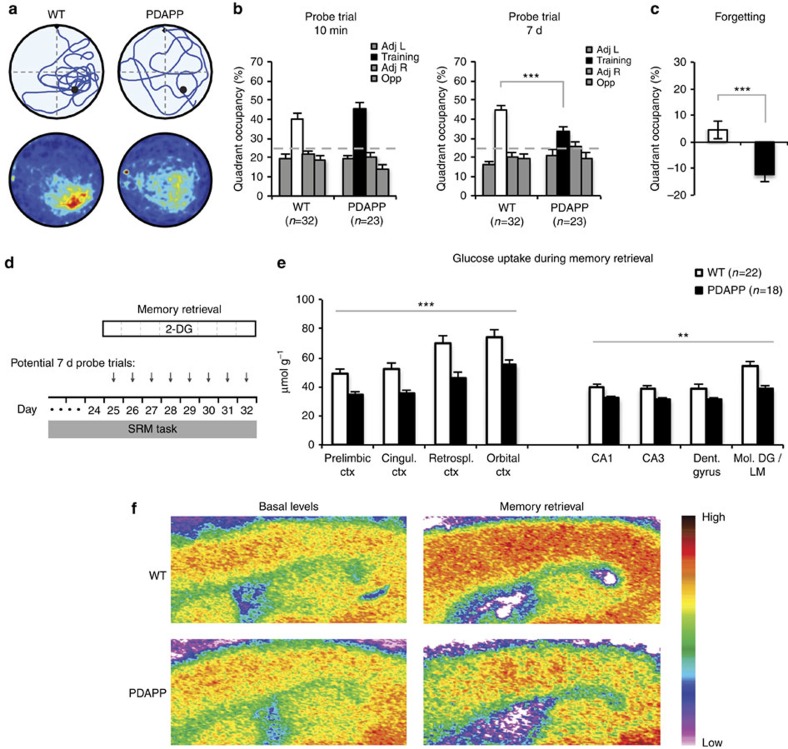
Impairments in memory retrieval and retrieval-associated glucose uptake in young PDAPP mice. (**a**) Swim path of representative individual animals (top) and group averaged heat-maps (bottom) for probe test at 7 days; only WT mice show a focus on the correct target location. (**b**) Young PDAPP mice searched equally well as WT mice in the training quadrant of the watermaze relative to the other quadrants in the probe trial 10 min after reaching the pre-defined criterion (ANOVA, genotype × quadrant: F_2,110_=1.18, *P*=0.31), but displayed significantly less focused searching at 7 days (genotype × quadrant: F_2,120_=4.05, *P*=0.016). The triple interaction of groups × quadrants × memory-delay was highly significant (F_2.5,134_=6.32, *P*=0.001, Greenhouse Geisser correction). A planned orthogonal comparison of training quadrant occupancies by WT and PDAPP mice at 7 days also revealed a highly significant difference: F_1,134_=11.04, *P*<0.001). Note: The occupancies in the training quadrant are represented by white bars for WT mice and by black bars for PDAPP mice. (**c**) Change in target quadrant occupancy (7 days minus 10 min) shows modest consolidation in WT mice but forgetting in PDAPP mice (*t*_53_=3.67, *P*<0.001). These data establish the greater forgetting by PDAPP mice over the 7-day (7d)retention period. (**d**) Experimental design incorporating possible time points for glucose uptake monitoring, given that learning criterion may be reached on any day between session 17 and 24. (**e**) Glucose uptake in the context of memory retrieval with injection of 2-DG immediately before the 7-d probe trial. Across midline structures in neocortex, a stronger metabolic signal was observed in WT mice than in PDAPP mice (ANOVA, between-subjects comparison: F_1,38_=14.25, *P*=0.001). A significant but smaller difference was observed for hippocampus: F_1,38_=7.53, *P*=0.01). (**f**) Representative pseudo-colour 2-DG images of brain sections from mice of the basal levels group (left) and the memory retrieval group (right) show the increase in metabolism in WT mice at retrieval and its absence in PDAPP animals, this difference more evident in the neocortex than in the hippocampus. Note: The scale bar at the side of the image serves the sole function of indicating that ‘warm' colours represent high 2-DG levels, whereas ‘cold' colours represent low levels (see the Methods for explanation of quantification). **P<0.01, ***P<0.001. Means±1 s.e.m. Cingul., cingulate, Ctx, cortex; Retrospl., retrosplenial.

However, when these animals were subject to memory retrieval tests 10 min (short interval) and 7 days (long interval) after reaching the training criterion, differential rates of forgetting were unmasked. An ‘Atlantis' platform was used to avoid memory extinction^36^. The escape platform is mounted on a spindle that is initially at the bottom of the watermaze, and so unavailable for escape, but which can be raised to within 1.5 cm of the water surface after a pre-determined time period (in our protocol: 60 s; Supplementary Fig. 5). The control mice showed very good memory search performance at both the short- and the long-retention intervals (Fig. 2a,b). In contrast, the PDAPP mice showed good recall at 10 min but searched across large areas of the pool 7 days later, indicative of substantial forgetting (Fig. 2a,b). The analysis of variance (ANOVA) showed a highly significant interaction between Groups, Quadrants and Memory-delay (F_2.5,134_=6.32, *P*=0.001, Greenhouse Geisser correction; Supplementary Table 6). The path-lengths while swimming in the watermaze were equivalent (Supplementary Fig. 6); what changed over time was the loss of search focus by the PDAPP mice. Furthermore, a quantitative comparison of the target quadrant occupancy scores between the 10-min and the 7-day probe tests showed no decline in the performance of WT mice after 7 days, but a strong decline in PDAPP mice (Fig. 2c). Thus, young PDAPP animals are forgetful over days.

A novel feature of our design was that it offered the opportunity to examine brain glucose uptake during distinct phases of memory formation and retrieval. Analysis of glucose uptake during the final 7-day probe test (‘memory retrieval' group; Fig. 2d) revealed a highly significant genotype × brain-regions interaction (F_3.1/116.3_=6.58, *P*<0.001; Greenhouse-Geisser correction for multiple comparisons; Supplementary Table 1). This interaction took the form of WT mice mobilizing glucose availability in specific brain regions during memory retrieval after a delay in a manner that the PDAPP mice could not. Brain region specificity was also reported in normal mice in a study of glucose uptake during memory consolidation that identified the engagement of key hippocampal and cortical regions^25^. Based on a similar genotype × brain-regions interaction, we also decided to focus on changes in uptake in memory-processing regions such as mid-line cortical regions, namely the prelimbic, cingulate, retrosplenial and orbital cortices, as well as the hippocampus^25^,^26^,^37^. In contrast to basal levels, we observed a strong and highly significant impairment in glucose uptake in PDAPP mice during memory retrieval in the neocortex, and a smaller but still significant change in hippocampus (Fig. 2e). Representative autoradiographs for WT and PDAPP mice for both basal and memory retrieval conditions indicate a striking hypermetabolism in WT mice in association with retrieval, which was absent in PDAPP mice (Fig. 2f). We recognize also the involvement of other parts of the brain in the observed phenotype, possibly reflecting a more widespread activation induced by the ‘survival' nature of the task, but the brain-regions interaction indicates that differential uptake is not uniform across the brain.

### Memory encoding/consolidation and glucose uptake

**Figure 3:**
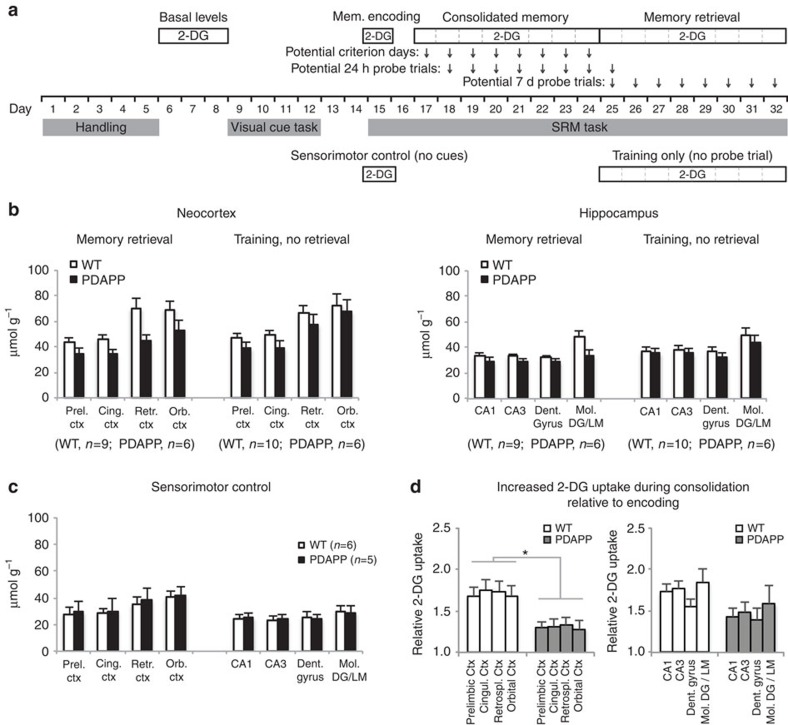
Alterations in glucose uptake reflect a deficit in memory consolidation in young PDAPP mice. (**a**) Experimental design showing different stages of training at which 2-DG monitoring was conducted and compared. The first day of spatial training was always session 15, but successive stages required the learning criterion to be met by individual animals. (**b**) Glucose uptake (i.v.) in the Memory Retrieval, and Training, No Retrieval sub-groups for neocortex (left) and hippocampus (right). The ANOVA showed an overall impairment of PDAPP relative to WT mice across the four neocortical regions (F_1,27_=4.23, *P*<0.05). There was a trend towards a greater difference if the Retrieval test was given, but the Genotype × Training Condition interaction was not significant (F<1), suggesting consolidation rather than retrieval as a major contributor to the retrieval-associated deficit. A similar trend observed in hippocampus did not reach significance for either comparison (F_1,27_=1.86, *P*>0.10; and F<1 respectively). (**c**) No difference was observed in glucose uptake between PDAPP and WT mice of the sensorimotor control group (ANOVAs: Fs<1). (**d**) Ratio of glucose-uptake (i.p.) values upon reaching criterion (as a measure of initial consolidation) relative to the first day of training (initial memory encoding, day 15), for the midline structures of the neocortex and hippocampus. The ANOVA showed a strong and significant difference between PDAPP and WT mice for neocortex (F_1,9_=8.38, *P*<0.02) but no significant difference for hippocampus (*P*>0.10). *P<0.05. Means±1 s.e.m. Cingul., cingulate, Ctx, cortex; Prel., prelimbic; Retrospl., retrosplenial.

We then investigated the cognitive specificity of this metabolic deficit in different steps addressing separate questions (Fig. 3). One issue has to do with memory consolidation being generally considered to be a slow process^37^. In our protocol, it is unlikely to be asymptotic by the time the mice reach criterion, and consolidation would be expected to continue throughout the 7-day post-training interval. Differences in glucose uptake during memory retrieval 7 days after reaching the criterion may therefore reflect group differences in either (i) memory retrieval processing or (ii) time-dependent consolidation, or possibly both processes. In additional trained groups of mice, we therefore compared glucose uptake during the 7-day probe test with that shown by a group given every aspect of the watermaze training up to but not including this final 7-day test, being injected with 2-DG in their cage at the equivalent time (the ‘training only' group; Fig. 3a). In this group, analysis of glucose uptake in the same mid-line structures of the neocortex implicated in memory consolidation showed smaller differences between PDAPP and WT mice compared with the ‘memory retrieval' group, although there was no significant difference between the patterns in the two groups (Fig. 3b left; and Supplementary Table 2). Similar but smaller trends in the same direction were observed in the hippocampus (Fig. 3b right; and Supplementary Table 2). The cautious interpretation is that memory consolidation over time is a major component of greater glucose uptake seen after 7 days in our WT mice, but the dual contribution of this and the act of memory retrieval may be required to see the full difference between WT and PDAPP mice in our 2-DG measures.

The second step involved analysis of glucose uptake in comparator groups at the start of spatial training before consolidation would have begun to take place. We compared a ‘basal levels group', a group given normal spatial training for one day (‘memory encoding'), and a ‘sensorimotor control group' yoked to the normal spatial training group in terms of mean escape latency for each of the six trials that day (with curtains excluding sight of extramaze cues to prevent spatial learning; Fig. 3a). All three groups showed similar patterns of glucose uptake across brain areas with no significant differences between PDAPP and WT mice (ANOVA, genotype × training condition: F<1; genotype × training condition × brain-region: F<1; Fig. 3c and Supplementary Tables 3 and 4). Note that swimming in the pool did cause slight reductions in core temperature, but the use of a heat lamp between trials ensured no major overall change in body temperature across all six trials that might have affected glucose uptake (Supplementary Fig. 7). These findings suggest that mere exposure to the watermaze (irrespective of training condition) and the process of encoding a new spatial memory affected glucose uptake in PDAPP and WT mice equivalently.

The third step addressed the gradual time course of the observed glucose-uptake change. If the differential glucose uptake seen in PDAPP mice at memory retrieval 7 days after reaching the criterion is partly due to the time-dependent process of memory consolidation, it should be possible to observe this develop during training by comparing the levels of glucose uptake on the first day of training with the levels seen upon reaching the training criterion (days 15 and criterion day; Fig. 3a). Comparison of these conditions revealed significantly higher glucose uptake in the neocortex in WT compared with PDAPP mice as criterion was reached (Fig. 3d left and Supplementary Table 4). A similar trend was observed for the hippocampal formation, but it did not reach significance, possibly because of a compensatory higher glucose uptake by PDAPP mice in hippocampus (Fig. 3d right and Supplementary Table 4). Given the absence of metabolic differences at the earliest stage of training, the developing phenotype as training proceeds might be a ‘signature' of metabolic abnormalities in the PDAPP mice that, in relevant brains areas, would contribute to impairment in systems consolidation.

### Rescue of the phenotypes by immunotherapy

Finally, we conducted preliminary experiments to explore whether these memory retrieval and glucose metabolism deficits could be rescued by immunotherapy with an antibody directed against part of the Aβ sequence^38^,^39^. The animal model we have used makes use of transgene overexpression that in theory might affect the phenotypes in a non-specific way related to the insertion of the transgene in the genome. An immunotherapy rescue experiment^38^,^39^ offers the opportunity to investigate the specificity of the phenotypes we have observed to Aβ and/or other APP metabolites containing the Aβ sequence^40^,^41^,^42^. Previous work using older mice at an age when they would normally have amyloid plaques indicated that preventive rescue can work behaviourally^43^,^44^. Our aim was to investigate whether immunotherapy could (i) also be effective in restoring memory function in animals that showed no change in the capacity to learn but only enhanced forgetting and (ii) rescue the metabolic deficits. The second of these is a study that, to our knowledge, has not been reported previously. As the number of animals that we have used in this part of our study was limited, these results are reported in the Supplementary Information.

Using young, pre-pathological PDAPP and WT mice, we examined the impact of weekly i.p. administration of the previously characterized 10D5 antibody against part of the Aβ sequence, compared with an isotype control antibody (TY11–15)^45^ on memory retrieval and retrieval-associated glucose uptake. As it was essential to have the antibody present during both training and the post-training period if its mode of action was to prevent a disruption of memory consolidation, we began administration 4 weeks before watermaze training (Supplementary Fig. 8a). Analysis of the 7-day probe test patterns revealed that treatment with 10D5 was successful in rescuing both the memory retrieval deficit in PDAPP mice and the memory-associated deficit in glucose uptake. Whereas PDAPP mice treated with the control antibody searched across larger areas of the pool, 10D5-treated PDAPP mice displayed a more focused search pattern in the target quadrant (Supplementary Fig. 8b). Quantitative analysis (ANOVA) revealed improvement induced by the 10D5 antibody, together with a significant difference in the target quadrant occupancy scores between 10D5-treated and control-treated PDAPP mice (Supplementary Fig. 8c). Swim path-lengths were equivalent across all four groups, suggesting no adverse effect of the 10D5 antibody on swimming (Supplementary Fig. 9). A separate indication of the effect by the 10D5 antibody in rescuing the forgetting phenotype involved comparing target quadrant occupancy scores at 10 min and 7 days in 10D5-treated and control-treated PDAPP mice (Supplementary Fig. 8d). Analysis of glucose uptake during the 7-day probe test revealed a significant effect of the 10D5 antibody in restoring levels of brain metabolism in PDAPP mice to those of WT mice, whereas this retrieval-associated glucose uptake in PDAPP mice treated with the control antibody was lower than that in WT mice (Supplementary Fig. 10 and Supplementary Table 5), as observed in our earlier experiments.

To investigate the possible mechanisms of action of 10D5, we also conducted ELISAs to measure the levels of soluble Aβ in the brain of the same mice. These confirmed that Aβ levels (both mouse and human Aβ) in PDAPP mice at this young age (4–5 months at the time of killing) were higher than endogenous levels in WT mice, but still relatively low (Supplementary Fig. 11). The data showed no overall difference in Aβ levels between 10D5-treated and control-antibody-treated PDAPP mice, suggesting initially that the mechanism of action of the 10D5 antibody might be by neutralizing the effects of Aβ rather than by altering its levels^46^. However, the ELISA measured both intracellular and extracellular Aβ, of which only the extracellular fraction is likely to compromise synaptic function^47^. We hypothesized that the antibody most likely acts on the much smaller extracellular fraction of soluble Aβ^48^, either by affecting Aβ levels (which would likely be undetected in the previous experiment) or by neutralizing Aβ without affecting its levels. To test this, we performed a separate experiment to measure extracellular Aβ in the interstitial fluid by microdialysis followed by ELISA. We used PDAPP mice treated with either 10D5 or the control antibody. Although a significant decrease was observed following 10D5, but not control treatment, compared with the basal levels average, this finding is not entirely conclusive as we did not observe a significant overall effect by ANOVA (Supplementary Fig. 12). These results cautiously suggest that the 10D5 antibody might act by lowering the levels of extracellular Aβ.

## Discussion

The main findings of this study are accelerated behavioural forgetting in young PDAPP mice in the absence of a learning deficit and an attenuation of memory-associated glucose uptake measured during the act of retrieval.

The novelty of our behavioural finding is that, in one prominent transgenic animal model of familial AD, we observe the paradoxical phenotype of accelerated forgetting of spatial memory in the absence of any learning impairment. This dissociation occurs at a very young age when hAPP transgenic mouse lines are generally considered normal. Murine behavioural models of AD generally look at learning rather than forgetting, and have largely focused on older ages when histological abnormalities are widespread. Often cognitive deficits are described in older mice, generally correlating with pathological abnormalities, but the explicit dissociation of distinct learning and memory mechanisms has not previously been explored. Our approach was to examine memory retrieval independently of spatial learning in the watermaze, by precisely matching the levels of original training across groups. Quite apart from the equivalence of learning, this enabled a definitive identification of early-stage forgetting detectable only over a long delay. A recent optogenetic study has, in parallel, provided evidence that forgetting in an animal model of AD may sometimes be due to retrieval failure^49^.

Interestingly, the second major finding of diminished memory-associated glucose uptake was also seen at an age when hAPP transgenic mouse models are generally considered presymptomatic. Changes in basal glucose uptake (that is, in animals that are not performing any task) have been observed at older, pathological ages in a hAPP mouse model^35^, but are barely detected in younger animals. Although we observed no alterations in glucose uptake during encoding or at criterion, a clear impairment was identified during delayed memory retrieval. In normal mice, changes in glucose uptake associated with memory consolidation have been previously reported^25^. It is therefore reasonable to propose that a contribution to the forgetting by our young PDAPP mice is a failure to meet consolidation-associated metabolic demands. Hippocampal inactivation in WT rats, using a GluR1–5 antagonist during the post-learning consolidation period, is associated with a 23% reduction of glucose uptake in the dorsal hippocampus and that it causes forgetting^50^. One effect of abnormal Aβ levels in young PDAPP mice may include targeting the synaptic stabilization in neocortex relevant for memory persistence^51^.

Our immunotherapy study was, unfortunately, conducted with too few animals for full statistical confidence. Nonetheless, using a full ANOVA rather than separate *t*-tests, we did observe a clear interaction-effect indicating that chronic treatment with an antibody directed against part of the Aβ sequence might protect against faster forgetting in young PDAPP mice and rescue retrieval-associated disturbances of glucose uptake. To our knowledge, our study is the first to demonstrate rescue of a glucose metabolism phenotype by Aβ-related immunotherapy. The behavioural finding extends a previous report showing a deficit and rescue of context fear conditioning in a different hAPP mouse model at the age of 5 months^45^, to which is now added that therapeutic rescue can also be seen at the most cryptic stage of the developing phenotype when learning is normal. Our findings are also fully consistent with our earlier observation with PDAPP mice that early immunotherapy can reduce levels of Aβ in a sub-set of mice to a point where, by way of association, learning and memory is normal^52^. However, the biochemical mechanisms of action of the 10D5 antibody remain largely unclear. The antibody we used targets all forms of Aβ, and it would therefore be of interest, in future work, to explore whether an antibody that specifically targets oligomeric Aβ, such as A887755 (ref. 53), would alleviate spatial memory forgetting given its effectiveness in rescuing novel object recognition memory in older hAPP mice^53^. It should also be acknowledged that the phenotypes that we observed in PDAPP mice might be mediated (either solely or partially) by other APP metabolites containing the Aβ sequence, in addition to Aβ itself, given the role of such metabolites in phenotypes of other hAPP transgenic mouse models^40^,^41^,^42^. Given the role of mitochondria in neuronal glucose metabolism and that mitochondrial transport is hindered by Aβ^54^, the cellular mechanisms underlying our observed phenotypes might include impaired synaptic localization of mitochondria.

The translation of animal data to human studies must always proceed with caution. The CANTAB test battery represents one systematic attempt to do this (http://www.cambridgecognition.com/academic/cantabsuite/tests) with the recent application of human ‘virtual' watermaze tasks to diagnosis in mild cognitive impairment (MCI)/AD being another^55^. If the idea emerging from our data can tentatively be extrapolated to humans, it raises the possibility that the diagnostic application of behavioural paradigms that examine memory retrieval after a longer delay than has been used to date, in conjunction with glucose uptake imaging (by fluorodeoxyglucose-positron emission tomography (FDG-PET)^56^) or functional magnetic resonance imaging^57^ might together serve as highly sensitive biomarkers of the cryptic but deleterious effects of Aβ long before even an MCI diagnosis. It is noteworthy that in our study glucose uptake at basal levels was found normal, the equivalent of a negative imaging diagnosis in humans. Further, we found that PDAPP mice performed very well in a memory retrieval test after a short delay—analogous to a negative diagnosis in an MCI test in which memory is tested after only a short delay. Thus, delay may be a critical parameter to vary to establish incipient problems. Interestingly, healthy young adults at genetic risk for AD have been recently shown to exhibit impaired spatial navigation and grid-cell-like representations^58^, further supporting the use of spatial memory paradigms for the earlier diagnosis of AD. It has also not escaped our notice that studies with presymptomatic subjects carrying familial AD mutations, examining episodic memory using delayed story recall, have revealed abnormalities up to 10 years before expected symptom onset^3^,^59^. Collectively, these findings point to the need to think beyond presently used cognitive testing paradigms to ones incorporating delayed testing.

There has been controversy over the apparent partial failure of immunotherapy in trials of AD patients^60^,^61^. However, the design of these clinical trials has been complicated, to date, by the need to enroll patients at a well-developed stage of the disease when treatment may already be less effective^62^. This has prompted widespread discussion of the desirability of moving towards earlier trials, such as those of presymptomatic individuals carrying mutations for familial AD^10^. It is noteworthy that the results of a recent anti-Aβ immunotherapy clinical trial, focusing on the subgroup of patients with mild AD, support a positive effect for early initiation of treatment^63^. The present data are consistent with the view that treatment should start as early as possible in AD, and they suggest that sensitive biomarkers that combine cognitive and physiological measures could be used towards that goal.

## Methods

### Subjects

A total of 184 hemizygous PDAPP transgenic (*n*=87) and littermate WT (*n*=97) female mice were used as subjects (including both watermaze-trained and untrained groups), derived from sublines 2,613 and 6,648. In the PDAPP mice, hAPP bearing the familial AD Indiana (V717F) mutation is overexpressed under the control of the platelet-derived growth factor-β promoter^12^. PDAPP and WT mice were supplied by the Janssen Alzheimer Immunotherapy, group housed (around 4 mice/cage) with free access to food and water, in a 12-h light/12-h dark cycle, adapted to the vivarium, checked for veterinary health and then subject to experimentation. The cohorts were derived from a breeding scheme involving breeding of the original PDAPP line with B6D2F1 mice (F1 generation of C57BL/6 and DBA/2 mice) and then breeding of the offspring with Swiss Webster mice. The subline 6,648 mentioned above is more recent, a subline started from cryopreserved embryos and involving the same breeding scheme. The use of the two sublines in our study has been carefully controlled and all mice used in any given experiment of our study (including both transgenic and WT mice) were always from the same subline. Also, all control groups were part of the same cohorts as the experimental groups that they were compared with (for example, 10D5 treatment versus control-antibody treatment) control for any instability of the PDAPP phenotype over time. We occasionally observed hyperactivity of some mice in the cage, but we did not observe any such behaviour in the watermaze. A small number of animals had seizures and these were excluded from the study. All animal experiments adhered to the UK Home Office ethical regulations of animal experimentation (Animals (Scientific Procedures) Act 1986).

### Watermaze

The watermaze was 1.96 m diameter, filled with water (25±2 °C) made opaque with white latex liquid, placed on a stand such that the water surface was at waist height, and situated within a room with numerous three-dimensional cues to enable effective spatial orientation^29^. The hidden ‘Atlantis' platform (diameter 12 cm; Supplementary Fig. 5) was used throughout the spatial reference memory task and was normally situated such that its top surface was 1.5 cm below the water surface, but could be secured on its spindle by electromagnets 25 cm below the water for probe trials before release after 60 s. It occupied a fixed location for each mouse, counterbalanced across locations within the pool within each group. For the visual cue task, a larger platform (diameter 20 cm) was used and it could be made visible by placing an object on its top surface. White curtains hanging from the ceiling could be pulled around the circumference of the pool to occlude extramaze cues during the visual cue task and during the equivalent of the first day of spatial learning in the sensorimotor control group.

### Behavioural protocols

All behavioural procedures were conducted blind such that the experimenter (V.B.) did not know the genotype (PDAPP or WT) or, when administered, the active or control immunotherapy antibody (10D5 or TY11–15, respectively). The behavioural protocols were based on those of our previous study with PDAPP mice^32^ with some modifications, as described in detail below. *Gentle handling* took place before training, during which the animals were calmed for 2–3 min per day for 5 days. *Visual cue training* involved use of the occluding curtains, 4 trials per day, for 4 or 5 days, and a changing location of the platform across trials. The animals were placed into the pool at randomly chosen start locations, were allowed to search for the platform for a maximum of 90 s and at the end of the trial were allowed to stay on the platform for 30 s. *Spatial training* involved pulling back the curtains to make the three-dimensional cues visible, 6 trials per day, 5 min inter-trial interval and 90 s maximum search time followed by 30 s time on the platform, for 3–10 days until the criterion of a daily session with an average escape latency<20 s was reached. In the case of the group of mice analysed with 2-DG upon reaching the criterion (‘Consolidated memory' group), training continued for one more day following reaching the criterion, as it could not be previously known on which day each animal would reach the criterion, in order to time the 2-DG administration. *Sensorimotor control* mice, following handling and training in the visual cue task as all other groups, were matched to the memory encoding group for trial duration and staying on the platform (by the use of the ‘Atlantis' platform) but had curtains around the pool and were therefore not trained with respect to the formation of spatial memory. *Probe tests* involved a single search trial lasting 60 s followed by release of the Atlantis platform to near (1.5 cm) of the water surface and additional time (30 s) to allow the mouse to find it. In the case of the 7-day probe test, the Atlantis platform was not used and the mice were removed from the pool after the end of the 60-s search period. In all training trials and probe tests, a paint roller was used to remove the mice from the pool and the mice were then transferred on the roller to a cage under a heat lamp for 5 min, to prevent hypothermia, before being transferred back to their home cage or subject to another trial. For the measurements of body temperature, subcutaneously injected transponders (Plexx, The Netherlands) were used. The number of animals in Fig. 1b is different compared with Fig. 1c–e, as the mice used in our ‘Memory Encoding' and ‘Sensorimotor Control' groups for 2-DG analysis (data presented in Fig. 3) were subjected to the full visual cue task (data presented in Fig. 1b), but only to one day of spatial training, that is, not to the full spatial training presented in Fig. 1c–e.

### Metabolic monitoring

[^14^C]-2-Deoxyglucose administration was by either an intravenous (i.v.) or an intraperitoneal (i.p.) route (see below). Mice were killed 45 min after the 2-DG injection by cervical dislocation (without perfusion) and the brain was dissected, frozen in −40 °C isopentane and stored at −80 °C until cryostat sectioning. A terminal blood sample was taken from each mouse and used for measurements of glucose and [^14^C] concentrations. 20 μm sagittal sections were cut through the right brain hemisphere of the killed mice in a cryostat. Each mouse that received an i.v. injection was placed in a restrainer and transferred to a heat chamber (30 °C) for 10 min (to achieve vein dilation) before the injection. Habituation to the restrainer and heat chamber took place for 3 days before the injection, with one session per day. Data in Fig. 1f were derived by assessing 2-DG uptake in WT (*n*=14) versus PDAPP (*n*=18) mice after the handling phase of the behavioural protocol without any watermaze training. Each animal was given 0.1 μmol per 5 μCi of 2-DG in 50 μl saline (by i.v. administration). During uptake, each animal was freely moving in the cage. Data in Fig. 2e and Supplementary Table 1 were derived by assessing 2-DG uptake in WT (*n*=22) versus PDAPP (*n*=18) mice. Each animal was given 0.1 μmol per 5 μCi of 2-DG in 50 μl saline (by i.v. administration). 25 s after the 2-DG injection, each animal performed a probe test in the watermaze (lasting 60 s), was transferred to a cage under a heat lamp for 5 min (freely moving) and then was transferred back to its home cage where it was freely moving. Identical conditions applied to the experiments presented in Fig. 3b and Supplementary Table 2 (only in the memory retrieval group; WT, *n*=9; PDAPP, *n*=6), in Supplementary Fig. 10 and Supplementary Table 5 (control antibody group; WT, *n*=8; PDAPP, *n*=6) and in Supplementary Fig. 10 and Supplementary Table 5 (10D5 antibody group; WT, *n*=5; PDAPP, *n*=4). Data in Fig. 3b and Supplementary Table 2 (training only group with No Retrieval) were derived by assessing 2-DG uptake in WT (*n*=10) versus PDAPP (*n*=6) mice. Each animal was given 0.1 μmol per 5 μCi of 2-DG in 50 μl saline (by i.v. administration). The 2-DG injection in this group of mice took place at the same time point as the memory retrieval group (7 days after reaching the criterion), but the mice remained in the cage (in the vivarium), freely moving, instead of being subject to a watermaze probe test. Data in Supplementary Table 3 (basal levels group) were derived by assessing 2-DG uptake in WT (*n*=6) versus PDAPP (*n*=4) mice after the handling phase of the behavioural protocol without any watermaze training. Each animal was given 0.1 μmol per 5 μCi of 2-DG in 400 μl saline (by i.p. administration). During uptake, each animal was freely moving in the cage. Data in Fig. 3c and Supplementary Table 3 (sensorimotor control group) were derived by assessing 2-DG uptake in WT (*n*=6) versus PDAPP (*n*=5) mice. Each animal was given 0.1 μmol per 5 μCi of 2-DG in 400 μl saline (by i.p. administration). Each animal was given the 2-DG injection before the watermaze trials of the equivalent of the first day of spatial training (see the Behavioural protocols) and was killed after the 6th trial of the same day. The six watermaze trials took place around the middle of the 45-min period between 2-DG injection and killing the mice, and the inter-trial interval was 5 min, with the mice remaining in a cage under a heat lamp between trials (freely moving). Identical conditions with respect to 2-DG monitoring applied to the experiment presented in Fig. 3d and Supplementary Table 4 (memory encoding group, denominator part in Fig. 3d; WT, *n*=4; PDAPP, *n*=3), with the only difference being that the cues in the watermaze room were visible and therefore proper spatial training was applied (see the Behavioural protocols). Identical conditions with respect to 2-DG monitoring also applied to the experiment presented in Fig. 3d and Supplementary Table 4 (consolidated memory group, numerator part in Fig. 3d; WT, *n*=6; PDAPP, *n*=5), in which the cues in the watermaze room were visible and therefore proper spatial training was applied, but in contrast to the memory encoding group, in this group 2-DG injection took place one day after each mouse has reached the criterion in watermaze performance (see the Behavioural protocols). The comparisons between different groups mentioned in the text are always between groups with the same type of 2-DG injection (i.v. or i.p.). In all the watermaze-trained groups receiving an i.p. injection of 2-DG, the mice performed six trials in the watermaze (with 5 min inter-trial interval), and the type of injection was chosen to be i.p. (which has relatively slow kinetics) in order for the uptake of 2-DG in the brain to coincide as closely as possible with performance in the watermaze. In all the watermaze-trained groups receiving an i.v. injection of 2-DG, the mice performed only one trial in the watermaze (a probe test), immediately (25 s) following 2-DG injection, therefore timing the peak of 2-DG availability (30 s post-injection when the i.v. route is used) with the act of memory retrieval. The final concentration of 2-DG in the blood was found to be ∼0.5% of the physiological blood glucose concentration, based on our measurements of blood glucose at the terminal sample (the figure of ∼0.5% was not used in the analysis in any quantitative way, and we provide this supplementary figure solely for the purpose of indicating that the injection of 2-DG involved only low (tracer level) concentrations, and it was therefore very unlikely to have any significant impact on the nutritional resources of the animal). Following autoradiographic exposure of films by the sections for 7 days, local optical density values (reflecting glucose uptake) were determined in 32 brain structures using an MCID image analysis system. These were transformed to radioactivity values (per gram tissue) by the use of standards of known amounts of radioactivity included during autoradiography, and normalized by multiplying by the concentration of glucose in the terminal blood sample and dividing by the concentration of [^14^C] in the same sample. The normalization procedure is based on the approximation that the radioactive signal is inversely proportional to the combined blood concentration of non-radioactive, physiological glucose plus 2-DG, and proportional to the [^14^C] concentration in the blood^64^. All 2-DG analyses were performed by the same experimenter (V.B.), who was also ‘blind' to the genotype and experimental group.

### Immunotherapy

Immunotherapy against the impact of elevated Aβ in PDAPP mice was conducted using a 2 × 2 design with four groups of mice representing the four combinations between the two genotypes (PDAPP and WT) and the two antibodies used (anti-Aβ antibody: 10D5; and isotype control antibody: TY11–15). Treatment took place by weekly i.p. administration of the antibody in PBS (dose: 10 mg kg^−1^), beginning 4 weeks before watermaze training and continuing through training.

### Immunohistochemistry

Overexpression of hAPP in the brain of PDAPP mice was confirmed by immunohistochemistry using sections that had been previously used for 2-DG autoradiography and an anti-APP antibody (8E5), confirming the correct genotype assignment of the mice. The absence of Aβ plaques in the brain of PDAPP mice at the upper end of the age range at the time of killing (5 months) was confirmed by immunohistochemistry using an anti-Aβ antibody (3D6). Immunohistochemical procedures took place using standard protocols.

### ELISA

The concentrations of TBS-soluble Aβ40 and Aβ42 in the brain of PDAPP and WT mice (using the other hemisphere from that used for 2-DG analysis) were determined by sandwich ELISA (Wako Pure Chemical Industries), according to the manufacturer's instructions. Brains were homogenized in cold TBS, including protease inhibitors, and homogenates were centrifuged in a microcentrifuge at maximum speed (supernatant recovered). BNT77 was used as the capture antibody for both Aβ40 and Aβ42, recognizing amino acids 11–28 of both human and mouse Aβ. The detection antibodies used were BA27 for the Aβ40 assay (recognizing the C-terminus of Aβ40) and BC05 for the Aβ42 assay (recognizing the C-terminus of Aβ42).

### Microdialysis

*In vivo* microdialysis sampling of brain interstitial fluid Aβ was performed as described previously^65^. Briefly, the microdialysis probe had a 4-mm shaft with a 3.0-mm, 1,000-kDa molecular weight cut-off (MWCO) polyethylene membrane (PEP-4-03; Eicom). Before use, the probe was conditioned by briefly dipping it in ethanol, and then washed with an artificial cerebrospinal fluid perfusion buffer containing 0.15% BSA that was filtered through a 0.2-μm-pore membrane. For probe implantation, the animals were anaesthetized with isoflurane, while a guide cannula (PEG-4; Eicom) was stereotactically implanted in the hippocampus (bregma −3.1 mm, −2.5 mm lateral to midline, −1.0 mm ventral to dura). The guide was fixed using binary dental cement. At 3 days after the implantation of the guide cannula, the mice were placed in a standard microdialysis cage, and a probe was inserted through the guide. After insertion of the probe, in order to obtain stable recordings, the probe and connecting tubes were perfused with artificial cerebrospinal fluid for 240 min at a flow rate of 10 μl min^−1^ before sample collection. Samples were collected at a flow rate of 0.5 μl min^−1^ and stored at 4 °C in polypropylene tubes during collection overnight. During microdialysis sample collection, mice were awake and freely moving in the microdialysis cage, designed to allow unrestricted movement of the animals without applying pressure on the probe assembly (AtmosLM microdialysis system; Eicom). Samples were first taken at basal levels (before antibody treatment) and after 48 h following a single dose of antibody treatment (10 mg kg^−1^, i.p.). Antibody treatment took place directly after collecting the basal level sample. Samples were analysed by ELISA as described in the previous section. Mice were 5 months old at sampling. Levels of interstitial fluid Aβ40 were not possible to analyse (only levels of Aβ42 were analysed) because of the assay used not being sensitive enough.

## Additional information

**How to cite this article:** Beglopoulos, V. *et al*. Early detection of cryptic memory and glucose uptake deficits in pre-pathological APP mice. *Nat. Commun.* 7:11761 doi: 10.1038/ncomms11761 (2016).

## Supplementary Material

Supplementary Information Supplementary Figures 1-12, Supplementary Tables 1-6 and Supplementary References.
