# Supplementary material for: Early detection of cryptic memory and glucose uptake deficits in pre-pathological APP mice
Source: Nat Commun. 2016 Jun 1;7:11761. doi: 10.1038/ncomms11761 (PMC4895343; doi:10.1038/ncomms11761)
Supplement: Supplementary Information — Supplementary Figures 1-12, Supplementary Tables 1-6 and Supplementary References. [file ncomms11761-s1.pdf]

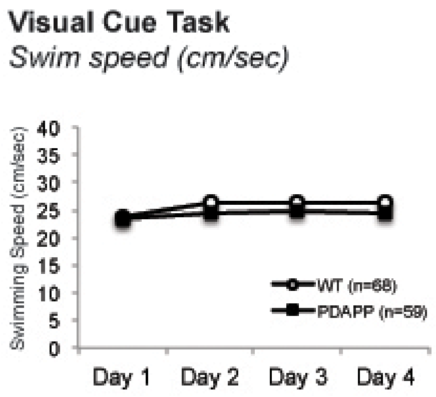

Supplementary Figure 1: *PDAPP mice swim in the watermaze normally.* Young WT and PDAPP mice show equivalent swim speed in the visual cue task (comparison between genotypes, ANOVA: $F_{1,125}$=3.14, p=0.08). Means +/- 1 S.E.M.

Contstruction of a “backward learning curve”

Escape latency across sessions (sec)

Mouse 1:	51	35	21	19	
Mouse 2:	63	25	17		
Mouse 3:	35	32	25	22	18

Backward learning curve

Mouse 1:		51	35	21	19
Mouse 2:			63	25	17
Mouse 3:	35	32	25	22	18
Backward learning curve (means):			**41**	**23**	**18**

Supplementary Figure 2: *The concept of a backward learning curve.* In the spatial learning version of the task, we checked on the rate of acquisition using a variety of standard and non-standard measures one of which is a ‘backward learning curve’(*1*). A backward learning curve is one that starts on the session on which criterion is actually reached by each individual animal (the criterion was a daily session of 6 trials with average escape latency <20 sec) and works back from there. Here the fictional data for escape latency of 3 individual animals is shown as normally collected across successive sessions (left), which is then averaged in alignment with the session at which criterion is reached (right)**.** The value of a backward learning curve is that it displays the pattern at which the criterion is reached across animals.

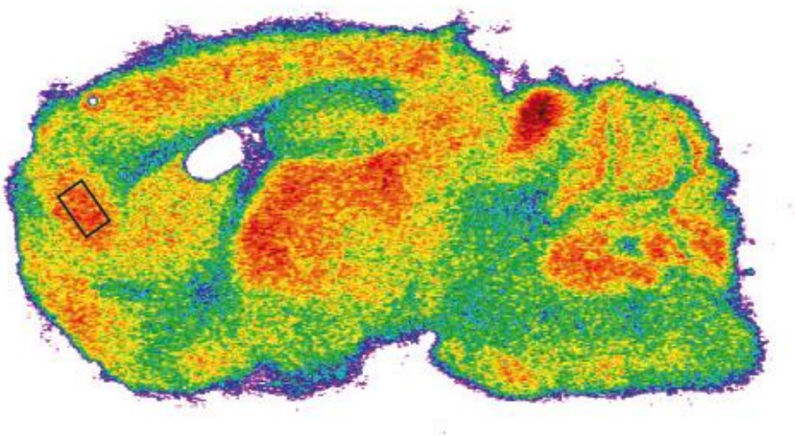

Supplementary Figure 3: Example of a pseudocolour [^{14}C]-2-deoxyglucose (2-DG) image of a mouse brain sagittal section. This shows, by way of example, a ‘box’ placed on the region of the orbital cortex (left part of image). Densitometric measurements were made (blind) in this and a total of 32 separate brain areas across multiple sections.

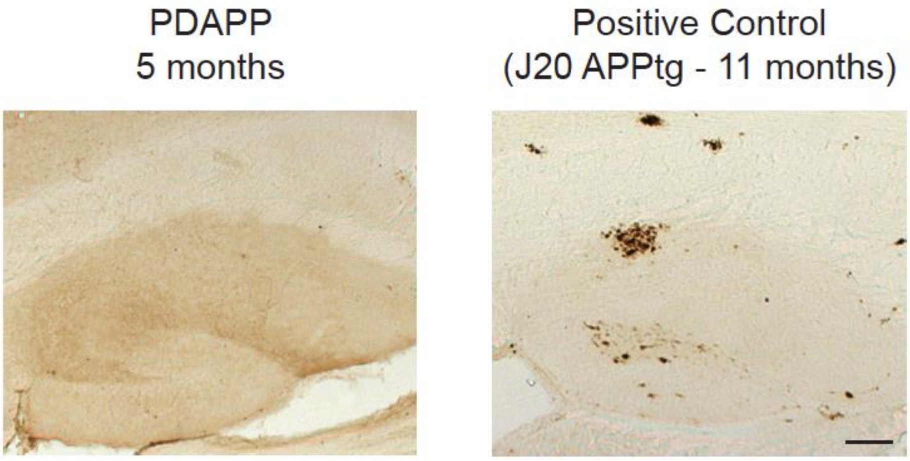

Supplementary Figure 4: *Absence of amyloid plaques in the brain of young PDAPP mice. Left:* Immunohistochemistry using the anti-Aβ antibody 3D6 confirmed the absence of amyloid plaques in the brain of PDAPP mice at 5 months of age (the oldest possible age at the end of all experiments; the mice were 3-4 months-old at the outset and the experiments lasted around 1 month. Image shown here is for the hippocampus. *Right:* A brain section from an 11 month-old APP transgenic mouse (exhibiting amyloid plaques) immunostained with the same antibody and protocol serves as a positive control (right). Scale bar: 250 μm.

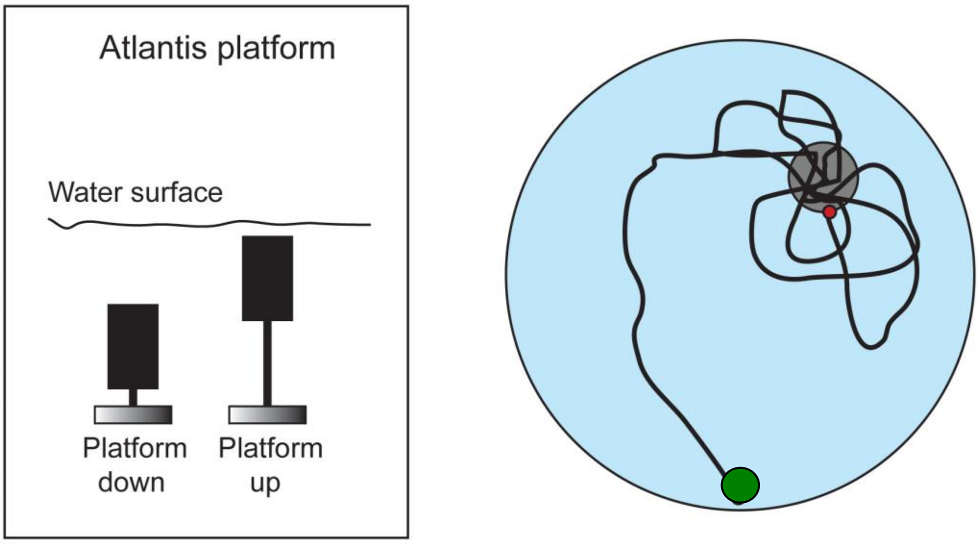

Supplementary Figure 5: *The Atlantis Platform in the watermaze*(*2*)*.* A cylindrical platform (made of Styrofoam) is held by electromagnets at the bottom of the pool (left). This prevents access to it and the swim paths can be across the correct location (right). When the platform is released, after 60 sec, it rises on a spindle to near the surface of the water enabling successful escape by the animals. This minimises memory extinction. The Atlantis Platform can be very useful if further within-subject probe tests are going to be performed. Green small circle – start of swim path; red small circle – end of swim path.

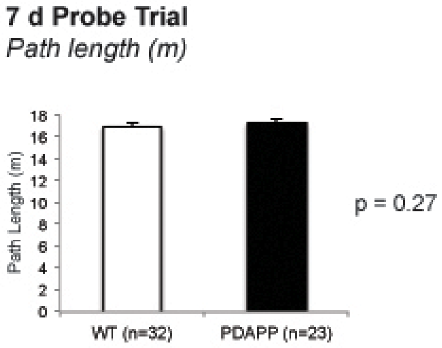

Supplementary Figure 6: *Normal search path-length in the watermaze by PDAPP mice.* The length of the swim path during the 7d memory retrieval probe test was equivalent between WT and PDAPP mice. The panels in the main manuscript show that the search pattern differed with respect to areas of the pool actually visited. Means +/- 1 S.E.M.

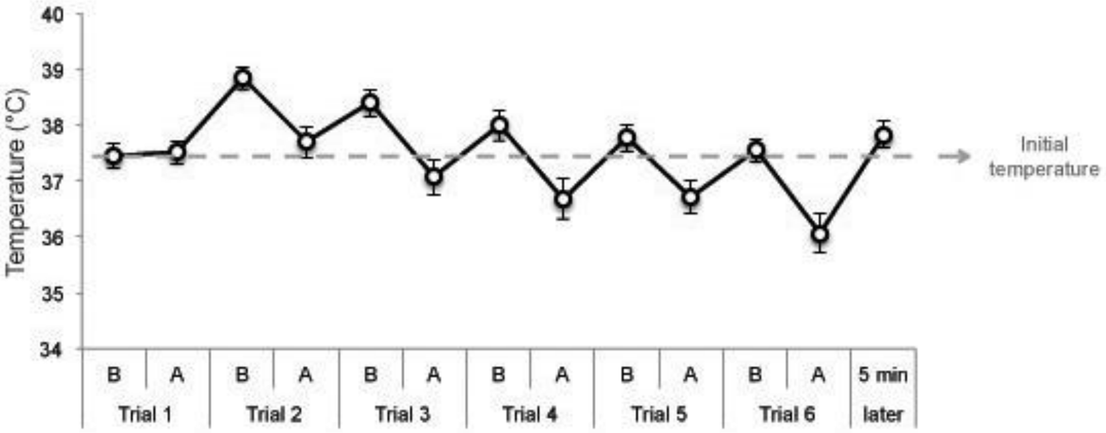

Supplementary Figure 7: Body temperature measurements before (B) and after (A) watermaze trials in a session of 6 trials matching the average swimming times of the memory encoding group, using identical conditions (including the inter-trial interval of 5 min) as in the rest of our study. Only minimal changes in body temperature were observed, with the temperature largely restored after each trial. There was no decrease in the body temperature after all 6 trials compared to that before the trials. These data suggest that it is unlikely that there are any major effects of body temperature on glucose uptake measurements. Means +/- 1 S.E.M.

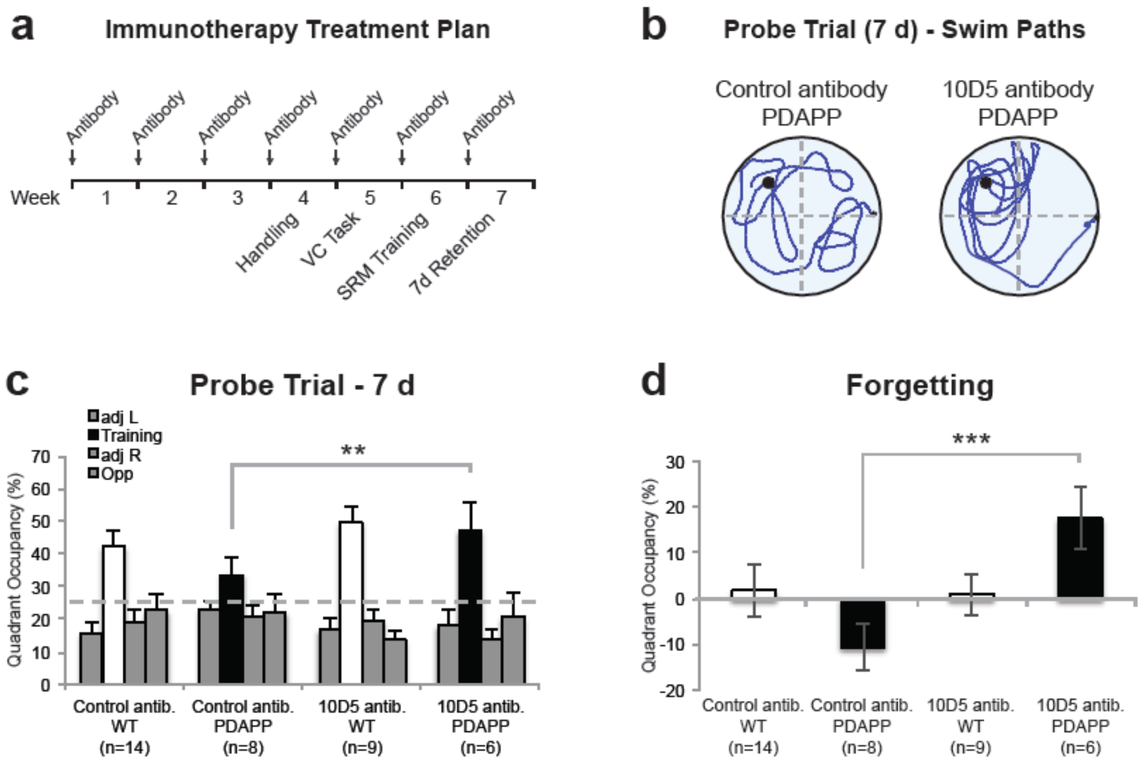

Supplementary Figure 8: *Rescue of spatial memory deficits in young PDAPP mice by anti-β-amyloid immunotherapy.* **A.** Immunotherapy regime, consisting of weekly i.p. administration of either the anti-Aβ (10D5) antibody or an isotype control antibody, beginning 4 weeks before watermaze training and continuing throughout training (dose = 10 mg/kg). **B.** Representative swim paths of 10D5-treated and control-treated PDAPP animals in the memory retrieval probe test 7 days after reaching criterion. **C.** Treatment with the control or the 10D5 antibody did not affect the probe test search pattern of WT mice at 7 d compared to that at 10 min (ANOVA, quadrants x memory-delay x antibody interaction: F<1). In contrast, a significant triple interaction was observed for the PDAPP mice ($F_{3,36}$=3.83, p=0.018). Data shown are scores at the 7 d probe test; a planned orthogonal comparison revealed significantly more focused searching in the target quadrant by PDAPP mice treated with 10D5 antibody than the control treatment ($F_{1,36}$=7.40, p=0.01; this contrast highlighted in figure). **D.** Change in target quadrant occupancy (7 days minus 10 min) shows no decline in WT mice (open bars) but rescue in PDAPP mice (ANOVA, genotype x antibody interaction, $F_{1,33}$=5.7, p=0.023; orthogonal comparison between the 2 PDAPP groups also reveals significant rescue: $F_{1,33}$=8.8, p<0.001). **: p≤0.01; ***: p<0.001. Means +/- 1 S.E.M.

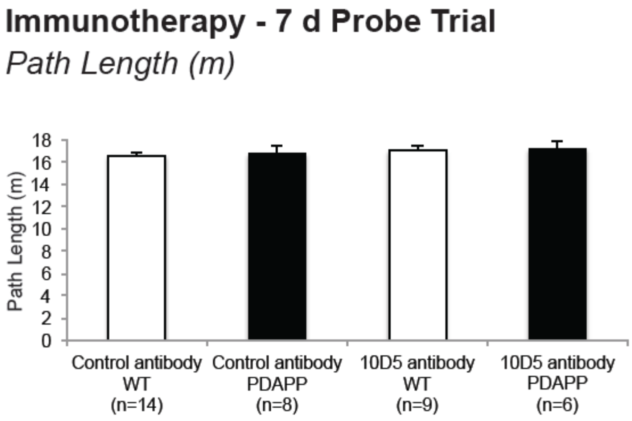

Supplementary Figure 9: *No adverse of the 10D5 anti-Aβ antibody on the search path-length in the watermaze.* The length of the swim path during the 7 d probe test was equivalent between mice treated with the 10D5 antibody (black) and mice treated with the control antibody (white). ANOVA: Fs<1 for a) genotype, b) treatment, and c) genotype x treatment comparisons. Means +/- 1 S.E.M.

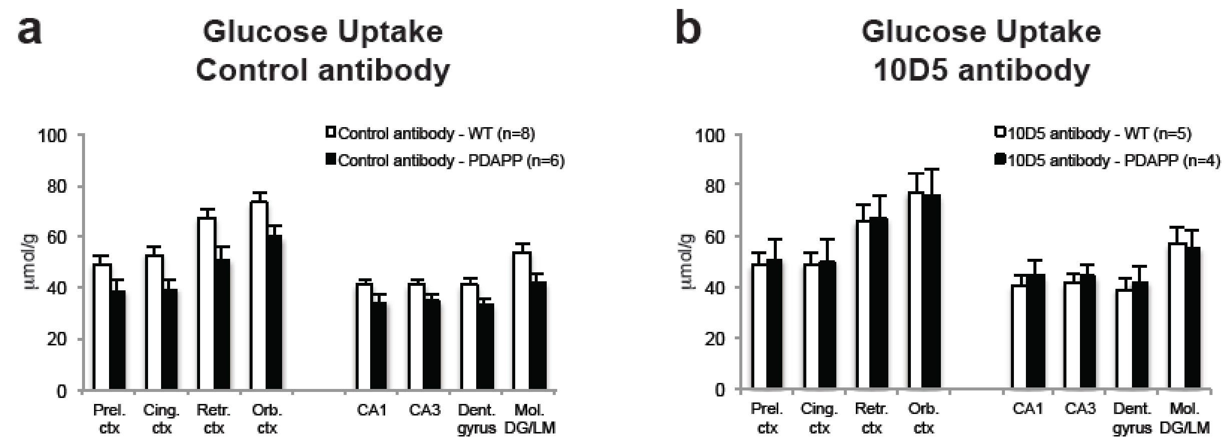

Supplementary Figure 10: *Rescue of memory retrieval-associated glucose uptake deficits in young PDAPP mice by anti-β-amyloid immunotherapy.* **A.** Analysis of glucose uptake in mice treated with the control antibody replicated the result of higher uptake in neocortex of WT mice compared to PDAPP mice during memory retrieval at 7 d (Neocortex: $F_{1,12}=7.22$, $p<0.025$; Hippocampus: $F_{1,12}=4.35$, $p=0.059$). **B.** Treatment with the 10D5 antibody rescued the impairment in glucose uptake in PDAPP mice during memory retrieval, with no genotype differences (Fs<1). Means +/- 1 S.E.M.

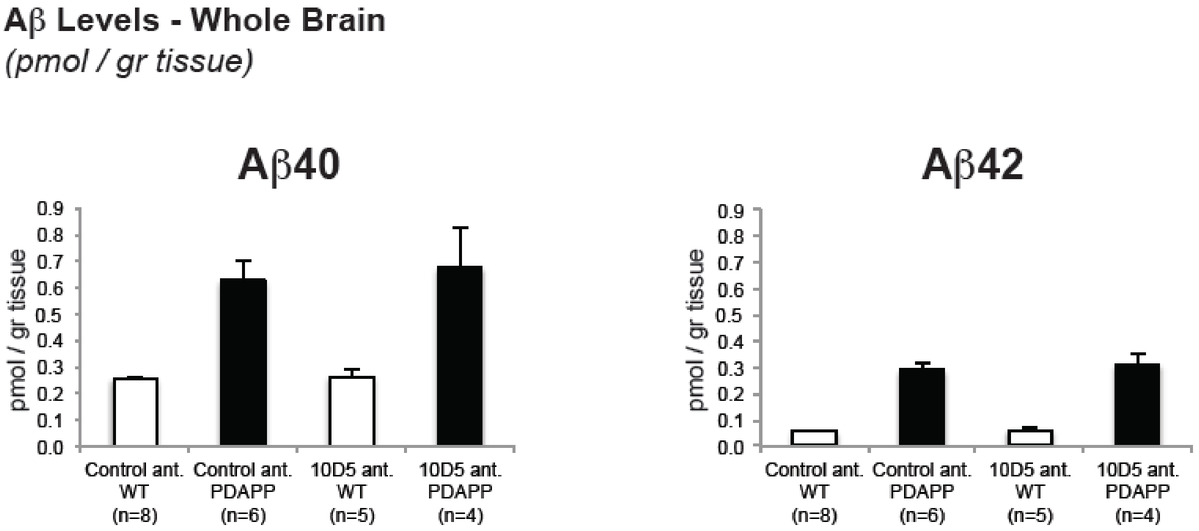

Supplementary Figure 11: *ELISA measures of soluble Aβ40 (left) and Aβ42 (right) across genotypic and immunotherapy treatment groups.* No difference was observed between 10D5-treated and control-antibody-treated PDAPP mice. ANOVAs showed higher levels in PDAPP compared to WT mice, as expected (Aβ40: $F_{1,19}$=37.2, $p<0.001$; Aβ42: $F_{1,19}$=145.1, $p<0.001$), but still relatively low at this young age. Means +/- 1 S.E.M.

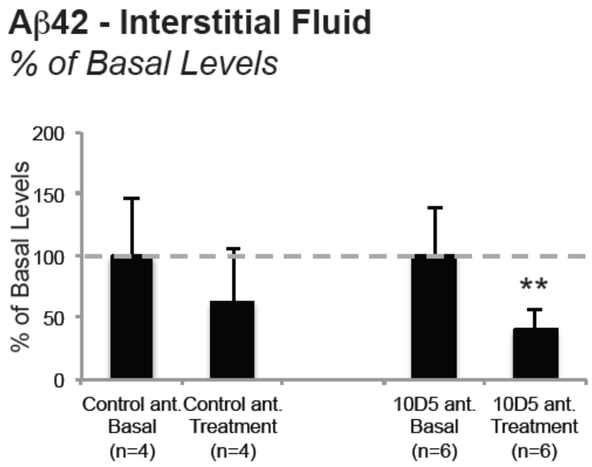

Supplementary Figure 12: *ELISA measures of Aβ42 in the interstitial fluid following microdialysis in PDAPP mice.* In a separate experiment from those described above, measures were taken of Aβ42 for each animal at basal levels (before antibody treatment) and after treatment. Values are expressed as % of the basal levels average for each treatment group. The dotted line indicates the basal levels average. An ANOVA showed a clear trend towards a decrease induced by the treatments ($F_{1,8}$=3.99, 0.1>p>0.05) but this did not differ across groups (F<1). However, the levels of Aβ42 after 10D5 treatment (but not control treatment) were significantly below the baseline average (dotted line). Aβ40 levels were not possible to analyse due to the assay used not being sensitive enough. **: p<0.01. Means +/- 1 S.E.M.

	WT (n=22)	PDAPP (n=18)
Prelimbic cortex	48.9 ± 3.3	34.6 ± 2.5
Cingulate cortex	52.1 ± 3.8	35.5 ± 2.2
Retrosplenial cortex	69.8 ± 4.9	46.5 ± 3.5
Orbital cortex	74.5 ± 4.8	54.9 ± 3.4
Frontal cortex	44.7 ± 2.4	36.4 ± 2.1
Motor cortex	59.3 ± 3.6	44.3 ± 2.7
Visual cortex	77.0 ± 5.9	62.0 ± 4.2
Somatosensory cortex	66.8 ± 4.0	50.9 ± 2.9
Caudate putamen	62.9 ± 3.7	49.1 ± 2.7
Nucleus accumbens	39.8 ± 3.2	28.3 ± 2.3
Globus pallidus	44.1 ± 2.7	33.7 ± 1.9
Lateral septum	39.5 ± 2.8	28.5 ± 1.6
Amygdala	36.1 ± 2.2	27.0 ± 1.5
Hippocampus (whole)	45.1 ± 3.0	33.8 ± 1.9
Cornu Ammonis 1	39.3 ± 2.6	32.0 ± 1.8
Cornu Ammonis 3	38.6 ± 2.4	31.3 ± 1.7
Dentate gyrus	39.2 ± 2.9	31.1 ± 1.6
Molec. DG / Lacun. mol.	54.0 ± 3.7	38.2 ± 2.3
Fimbria hippocampus	20.8 ± 1.3	15.7 ± 0.8
Dorsal subiculum	59.8 ± 4.0	42.8 ± 2.5
Postsubiculum	60.3 ± 3.7	41.5 ± 2.5
Thalamus (whole)	67.7 ± 4.0	49.3 ± 2.8
LP thalamic nucleus	77.3 ± 5.6	51.6 ± 3.4
L-D thalamic nucleus	73.1 ± 5.5	47.9 ± 2.9
D-L geniculate nucleus	85.0 ± 5.4	59.1 ± 3.8
Subthalamic nucleus	66.2 ± 4.0	48.5 ± 2.7
Zona inserta	65.2 ± 3.8	47.4 ± 2.5
Hypothalamus	35.3 ± 2.1	27.1 ± 1.6
Superior colliculus	55.1 ± 2.9	44.1 ± 2.2
Inferior colliculus	98.2 ± 5.7	78.0 ± 5.2
Periaquedactal grey	33.1 ± 2.4	27.4 ± 1.8
Cerebellum	55.8 ± 2.8	41.6 ± 2.3

Supplementary Table 1: Glucose uptake (μmol/g) in brain structures during memory retrieval after 7 days. Values are expressed as average ± SEM.

	Training + Memory Retrieval		Training Only	
	WT (n=9)	PDAPP (n=6)	WT (n=10)	PDAPP (n=6)
Prelimbic cortex	43.4 ± 3.6	34.5 ± 3.9	47.1 ± 3.2	38.7 ± 5.2
Cingulate cortex	45.3 ± 4.3	33.9 ± 3.6	49.0 ± 3.9	38.8 ± 5.4
Retrosplenial cortex	69.4 ± 8.7	44.2 ± 5.6	66.6 ± 5.9	56.8 ± 9.0
Orbital cortex	69.2 ± 6.7	53.1 ± 7.0	72.3 ± 9.4	67.0 ± 9.5
Frontal cortex	39.1 ± 3.0	34.9 ± 4.0	42.6 ± 4.4	39.8 ± 4.8
Motor cortex	53.5 ± 4.5	43.8 ± 5.4	55.3 ± 6.0	51.9 ± 6.7
Visual cortex	69.7 ± 6.3	62.0 ± 9.7	77.5 ± 10.7	78.0 ± 11.5
Somatosensory cortex	62.3 ± 5.4	48.9 ± 6.1	62.4 ± 7.3	60.0 ± 7.3
Caudate putamen	56.9 ± 4.4	45.5 ± 4.8	55.6 ± 6.0	53.6 ± 6.7
Nucleus accumbens	33.2 ± 2.2	24.2 ± 4.6	38.1 ± 3.1	28.3 ± 3.7
Globus pallidus	38.6 ± 2.6	30.5 ± 2.8	40.0 ± 4.2	33.8 ± 4.2
Lateral septum	34.8 ± 2.6	26.0 ± 2.9	36.9 ± 3.1	33.4 ± 4.5
Amygdala	30.3 ± 1.9	24.6 ± 2.4	36.3 ± 3.8	30.7 ± 3.6
Hippocampus (whole)	39.6 ± 3.0	31.1 ± 3.1	41.2 ± 4.4	37.7 ± 4.3
Cornu Ammonis 1	33.5 ± 2.3	29.2 ± 2.8	37.0 ± 3.9	35.3 ± 4.1
Cornu Ammonis 3	33.2 ± 1.8	28.7 ± 2.5	37.7 ± 3.9	35.2 ± 4.2
Dentate gyrus	32.1 ± 2.0	28.8 ± 2.5	36.6 ± 3.5	32.6 ± 3.5
Molec. DG / Lacun. mol.	48.2 ± 4.3	33.7 ± 3.8	49.9 ± 5.8	43.7 ± 5.7
Fimbria hippocampus	17.3 ± 1.2	14.8 ± 1.5	19.5 ± 2.2	15.8 ± 1.7
Dorsal subiculum	53.8 ± 4.2	39.1 ± 4.3	54.1 ± 5.8	45.4 ± 5.7
Postsubiculum	54.7 ± 4.1	38.0 ± 3.6	54.0 ± 5.7	45.7 ± 5.6
Thalamus (whole)	60.6 ± 4.2	46.6 ± 5.3	61.1 ± 6.2	52.3 ± 5.8
LP thalamic nucleus	70.8 ± 6.0	45.8 ± 5.3	67.0 ± 7.4	60.0 ± 7.8
L-D thalamic nucleus	66.2 ± 5.6	44.8 ± 5.3	64.8 ± 7.6	52.5 ± 6.5
D-L geniculate nucleus	78.2 ± 6.2	53.7 ± 6.1	79.6 ± 9.6	68.7 ± 9.9
Subthalamic nucleus	59.5 ± 5.1	43.8 ± 4.8	57.3 ± 6.6	48.3 ± 6.4
Zona inserta	60.9 ± 5.4	45.1 ± 4.9	57.9 ± 6.4	50.4 ± 6.5
Hypothalamus	29.8 ± 1.9	25.6 ± 2.7	32.6 ± 2.3	28.7 ± 3.4
Superior colliculus	54.2 ± 4.4	42.2 ± 4.6	51.0 ± 5.7	49.6 ± 5.8
Inferior colliculus	103.3 ± 10.2	80.8 ± 12.5	104.5 ± 13.4	97.0 ± 13.5
Periaqueductal grey	28.3 ± 2.1	24.8 ± 2.5	31.2 ± 2.7	27.1 ± 3.1
Cerebellum	52.9 ± 3.0	39.3 ± 4.2	50.8 ± 5.7	47.3 ± 7.0

Supplementary Table 2: Glucose uptake (μmol/g) in brain structures in the ‘training + memory retrieval’ group and the ‘training only’ group. The data of the training + memory retrieval group shown here are included in the combined data presented in Table 1. The two groups shown here were trained and analysed at the same time. Values are expressed as average ± SEM.

	Sensorimotor Control		Basal Levels (i.p.-injected)	
	WT (n=6)	PDAPP (n=5)	WT (n=6)	PDAPP (n=4)
Prelimbic cortex	27.7 ± 4.9	29.1 ± 8.6	32.1 ± 4.3	25.5 ± 0.8
Cingulate cortex	28.8 ± 3.4	30.2 ± 9.0	33.5 ± 4.2	25.0 ± 1.5
Retrosplenial cortex	35.1 ± 5.1	38.2 ± 8.7	40.1 ± 6.3	31.1 ± 1.4
Orbital cortex	40.1 ± 4.6	42.1 ± 6.1	50.4 ± 9.6	41.9 ± 1.9
Frontal cortex	26.8 ± 3.4	26.1 ± 3.4	28.3 ± 4.9	25.7 ± 0.9
Motor cortex	32.3 ± 4.1	33.8 ± 4.7	35.4 ± 5.8	31.3 ± 1.0
Visual cortex	41.2 ± 4.8	44.6 ± 7.9	53.5 ± 8.7	45.0 ± 1.3
Somatosensory cortex	35.2 ± 5.1	37.4 ± 5.3	39.9 ± 6.8	36.2 ± 1.1
Caudate putamen	36.5 ± 4.6	40.2 ± 6.0	36.3 ± 6.1	34.7 ± 1.1
Nucleus accumbens	25.8 ± 4.9	27.2 ± 5.2	22.6 ± 3.4	23.0 ± 1.5
Globus pallidus	26.4 ± 3.7	25.9 ± 3.6	27.4 ± 4.6	24.4 ± 0.9
Lateral septum	24.8 ± 3.3	27.4 ± 4.5	23.7 ± 3.3	20.9 ± 0.6
Amygdala	22.2 ± 3.1	23.9 ± 3.6	23.8 ± 4.0	20.5 ± 0.7
Hippocampus (whole)	26.8 ± 3.4	26.4 ± 3.9	30.5 ± 4.8	27.7 ± 1.8
Cornu Ammonis 1	24.1 ± 3.4	25.0 ± 3.7	27.1 ± 4.3	26.0 ± 2.2
Cornu Ammonis 3	23.5 ± 3.0	23.7 ± 3.6	26.9 ± 4.2	26.3 ± 1.6
Dentate gyrus	25.3 ± 4.0	24.4 ± 3.4	28.1 ± 4.5	25.7 ± 2.2
Molec. DG / Lacun. mol.	30.2 ± 3.3	28.7 ± 4.9	34.3 ± 5.8	31.5 ± 1.7
Fimbria hippocampus	15.3 ± 2.5	15.2 ± 2.0	15.0 ± 2.1	13.0 ± 0.4
Dorsal subiculum	34.3 ± 3.6	33.2 ± 5.2	37.7 ± 7.2	33.0 ± 1.2
Postsubiculum	35.6 ± 4.1	34.3 ± 5.3	34.0 ± 7.9	31.3 ± 0.7
Thalamus (whole)	39.8 ± 4.8	38.4 ± 5.3	38.2 ± 6.3	35.1 ± 0.7
LP thalamic nucleus	42.9 ± 4.4	40.9 ± 6.8	43.6 ± 7.3	38.7 ± 1.1
L-D thalamic nucleus	39.0 ± 4.3	38.2 ± 7.0	41.9 ± 7.2	37.3 ± 1.2
D-L geniculate nucleus	43.2 ± 5.1	43.4 ± 7.5	50.9 ± 8.2	43.7 ± 1.4
Subthalamic nucleus	40.0 ± 5.8	37.9 ± 6.0	38.7 ± 7.2	37.3 ± 1.5
Zona inserta	36.3 ± 4.8	34.6 ± 5.2	37.5 ± 6.5	34.4 ± 1.4
Hypothalamus	23.4 ± 5.1	23.2 ± 3.4	21.1 ± 3.0	22.2 ± 1.6
Superior colliculus	37.6 ± 6.1	35.5 ± 4.9	38.2 ± 8.2	34.7 ± 2.3
Inferior colliculus	58.0 ± 8.6	61.1 ± 9.5	69.8 ± 13.4	60.3 ± 5.7
Periaquedactal grey	27.7 ± 12.5	27.9 ± 4.2	21.5 ± 3.8	22.2 ± 1.4
Cerebellum	33.8 ± 9.5	34.4 ± 4.2	38.7 ± 7.1	31.0 ± 2.3

Supplementary Table 3: Glucose uptake (μmol/g) in brain structures in the sensorimotor control and basal levels groups (i.p.injected). Values are expressed as average ± SEM.

	Memory Encoding		Consolidated Memory	
	WT (n=4)	PDAPP (n=3)	WT (n=6)	PDAPP (n=5)
Prelimbic cortex	17.9 ± 1.4	20.6 ± 4.5	30.0 ± 1.9	26.7 ± 1.3
Cingulate cortex	18.5 ± 1.5	19.9 ± 4.3	32.4 ± 2.2	26.1 ± 1.9
Retrosplenial cortex	23.6 ± 2.2	25.7 ± 3.6	40.9 ± 2.7	34.2 ± 2.2
Orbital cortex	27.2 ± 1.2	30.6 ± 5.1	45.6 ± 3.4	39.0 ± 3.3
Frontal cortex	16.9 ± 1.4	22.0 ± 3.7	29.2 ± 1.7	25.6 ± 2.2
Motor cortex	19.8 ± 1.8	23.7 ± 4.3	34.7 ± 2.1	30.8 ± 2.1
Visual cortex	28.3 ± 2.7	35.7 ± 4.1	46.8 ± 3.5	40.1 ± 3.8
Somatosensory cortex	23.1 ± 2.5	25.8 ± 5.6	40.3 ± 2.7	35.7 ± 3.2
Caudate putamen	22.7 ± 1.7	27.5 ± 4.5	39.5 ± 3.0	35.4 ± 2.7
Nucleus accumbens	16.7 ± 1.1	17.0 ± 1.7	26.6 ± 2.2	23.8 ± 2.1
Globus pallidus	16.4 ± 1.4	16.6 ± 2.6	29.0 ± 1.9	24.2 ± 2.0
Lateral septum	15.5 ± 1.1	16.2 ± 3.2	27.0 ± 1.6	20.4 ± 1.2
Amygdala	14.0 ± 1.4	15.7 ± 1.3	23.7 ± 1.5	19.7 ± 1.7
Hippocampus (whole)	17.2 ± 1.4	18.6 ± 3.0	31.5 ± 1.7	27.1 ± 2.6
Cornu Ammonis 1	15.4 ± 0.8	17.3 ± 2.6	26.6 ± 1.4	24.7 ± 1.9
Cornu Ammonis 3	14.9 ± 0.6	16.1 ± 2.1	26.3 ± 1.5	23.9 ± 2.0
Dentate gyrus	17.6 ± 0.7	17.1 ± 2.0	27.2 ± 1.8	23.8 ± 2.4
Molec. DG / Lacun. mol.	20.6 ± 1.7	20.5 ± 4.3	38.0 ± 3.2	32.6 ± 4.6
Fimbria hippocampus	8.7 ± 1.3	8.0 ± 1.6	15.8 ± 1.3	12.7 ± 1.1
Dorsal subiculum	22.8 ± 1.7	22.4 ± 4.1	37.1 ± 2.4	32.6 ± 3.0
Postsubiculum	24.9 ± 1.5	22.1 ± 4.6	38.9 ± 3.2	33.6 ± 2.7
Thalamus (whole)	25.8 ± 1.9	27.9 ± 5.0	41.7 ± 2.7	35.2 ± 3.4
LP thalamic nucleus	30.2 ± 2.6	30.6 ± 5.2	47.5 ± 4.1	38.8 ± 4.1
L-D thalamic nucleus	28.2 ± 2.9	29.0 ± 5.3	44.1 ± 3.2	38.9 ± 4.3
D-L geniculate nucleus	31.6 ± 1.8	35.3 ± 5.3	51.7 ± 4.1	40.0 ± 4.2
Subthalamic nucleus	24.3 ± 3.9	28.6 ± 4.9	41.9 ± 3.4	35.8 ± 3.8
Zona inserta	21.9 ± 1.5	26.3 ± 3.8	38.9 ± 2.5	34.3 ± 3.1
Hypothalamus	13.5 ± 2.0	15.6 ± 1.6	25.5 ± 2.1	21.6 ± 2.0
Superior colliculus	22.0 ± 2.0	27.5 ± 2.4	38.9 ± 2.9	30.6 ± 2.1
Inferior colliculus	32.3 ± 1.2	46.0 ± 13.9	61.3 ± 6.6	52.3 ± 6.3
Periaquedactal grey	13.8 ± 1.9	19.7 ± 2.7	22.1 ± 2.2	20.4 ± 0.7
Cerebellum	20.5 ± 1.5	24.7 ± 5.3	33.6 ± 2.7	30.0 ± 2.1

Supplementary Table 4: Glucose uptake (μmol/g) in brain structures in the memory encoding and consolidated memory groups. Values are expressed as average ± SEM.

	Control antibody		10D5 antibody	
	WT (n=8)	PDAPP (n=6)	WT (n=5)	PDAPP (n=4)
Prelimbic cortex	49.3 ± 3.1	38.8 ± 3.8	48.6 ± 4.6	51.0 ± 7.9
Cingulate cortex	53.0 ± 3.3	39.7 ± 3.5	49.1 ± 4.7	50.3 ± 8.6
Retrosplenial cortex	67.1 ± 3.6	50.8 ± 5.0	65.7 ± 6.8	66.6 ± 9.1
Orbital cortex	73.2 ± 3.8	59.5 ± 5.0	77.0 ± 7.7	75.6 ± 11.0
Frontal cortex	48.5 ± 1.7	40.8 ± 3.0	46.6 ± 4.9	49.9 ± 7.7
Motor cortex	60.4 ± 3.4	49.1 ± 3.9	58.1 ± 7.0	63.1 ± 10.0
Visual cortex	73.2 ± 4.2	65.4 ± 4.9	75.8 ± 8.4	83.4 ± 12.9
Somatosensory cortex	67.6 ± 4.2	56.4 ± 4.3	69.1 ± 8.7	68.9 ± 10.1
Caudate putamen	65.0 ± 3.7	55.8 ± 4.2	65.9 ± 7.4	70.0 ± 10.6
Nucleus accumbens	39.4 ± 1.7	31.8 ± 2.8	40.0 ± 5.3	40.8 ± 6.2
Globus pallidus	46.9 ± 3.0	39.1 ± 3.6	46.3 ± 5.1	47.1 ± 6.9
Lateral septum	41.2 ± 2.6	31.7 ± 2.1	40.7 ± 6.1	42.2 ± 5.8
Amygdala	39.1 ± 2.5	31.4 ± 2.8	40.3 ± 5.0	39.2 ± 5.5
Hippocampus (whole)	46.0 ± 2.8	36.3 ± 3.1	46.1 ± 5.5	47.2 ± 6.5
Cornu Ammonis 1	41.0 ± 2.2	34.3 ± 2.9	40.5 ± 4.5	44.2 ± 6.4
Cornu Ammonis 3	40.9 ± 2.4	34.5 ± 3.1	41.5 ± 4.2	44.1 ± 5.2
Dentate gyrus	40.8 ± 3.2	33.0 ± 2.7	39.5 ± 4.5	42.1 ± 6.1
Molec. DG / Lacun. mol.	53.8 ± 3.0	41.9 ± 3.5	56.8 ± 6.4	55.3 ± 7.3
Fimbria hippocampus	22.9 ± 1.6	17.6 ± 1.5	21.4 ± 2.7	22.0 ± 2.6
Dorsal subiculum	59.1 ± 3.3	45.7 ± 4.0	58.6 ± 7.1	56.2 ± 7.4
Postsubiculum	59.8 ± 3.9	45.1 ± 4.0	61.5 ± 7.9	55.0 ± 7.7
Thalamus (whole)	70.0 ± 3.6	56.0 ± 4.2	70.7 ± 8.5	70.2 ± 12.3
LP thalamic nucleus	75.1 ± 3.6	58.8 ± 4.9	77.7 ± 9.4	71.2 ± 10.7
L-D thalamic nucleus	72.0 ± 3.5	52.3 ± 3.8	71.5 ± 8.1	66.3 ± 9.6
D-L geniculate nucleus	84.1 ± 4.0	67.5 ± 5.4	86.8 ± 12.1	83.3 ± 13.9
Subthalamic nucleus	66.6 ± 3.5	53.5 ± 4.4	68.4 ± 7.4	67.6 ± 11.3
Zona inserta	66.2 ± 3.3	53.3 ± 4.1	67.3 ± 7.2	66.1 ± 10.4
Hypothalamus	38.0 ± 2.2	30.6 ± 3.6	36.3 ± 4.4	40.0 ± 6.3
Superior colliculus	55.3 ± 3.0	46.5 ± 3.7	53.0 ± 6.1	57.8 ± 8.5
Inferior colliculus	91.9 ± 4.1	85.4 ± 7.0	99.6 ± 12.9	104.9 ± 11.8
Periaquedactal grey	36.4 ± 2.2	29.1 ± 2.8	39.0 ± 4.6	40.9 ± 5.9
Cerebellum	58.4 ± 3.3	46.5 ± 4.3	62.9 ± 6.2	60.5 ± 8.6

Supplementary Table 5: Glucose uptake (μmol/g) in brain structures during memory retrieval after 7 days in the control antibody-treated group and the 10D5 antibody-treated group. Values are expressed as average ± SEM.

Supplementary Table 6: Full quadrant scores for the 10 min and 7 d probe tests.

WT 10 min

Animal	Quadrant time (%) adj L	Training	adj R	Opp
F2711	8.54	34.17	26.80	30.49
F2715	14.43	41.28	27.69	16.61
F2719	9.48	68.22	22.30	0.00
F2720	7.16	47.59	31.28	13.98
F2843	10.39	40.54	31.83	17.25
F2842	14.93	53.86	15.27	15.94
F2847	8.22	76.85	14.93	0.00
F2848	29.65	29.15	25.29	15.91
F2850	14.74	55.95	20.94	8.38
F4512	2.01	30.32	42.71	24.96
F4513	20.63	40.77	25.46	13.15
F4527	52.16	8.97	0.00	38.87
F4528	27.69	34.06	23.15	15.10
F4535	34.11	22.63	16.47	26.79
F4536	13.81	34.11	14.81	37.27
F4567	29.65	40.20	16.92	13.23
F4568	41.76	27.12	12.65	18.47
F4587	16.11	32.06	41.20	10.63
F5714	25.58	30.40	15.28	28.74
F5715	17.94	25.08	31.56	25.42
F5733	2.33	46.26	26.29	25.13
F5734	16.14	21.30	35.11	27.45
F5740	11.96	73.26	13.62	1.16
F6302	37.21	34.05	12.79	15.95
F6308	32.05	34.73	15.60	17.62
F6310	25.67	29.03	7.22	38.09
F6324	7.04	73.20	5.70	14.07
F6325	22.95	29.98	18.59	28.48
F7503	34.17	21.94	21.94	21.94
F7513	11.96	40.86	23.92	23.26
F7520	8.54	54.77	25.46	11.22
F7523	11.58	51.85	25.67	10.91
**Average**	**19.39**	**40.14**	**21.51**	**18.95**
SE	**2.15**	**2.90**	**1.70**	**1.80**
SQRT(N)	5.66	5.66	5.66	5.66

WT 7 days

Animal	Quadrant time (%) adj L	Training	adj R	Opp
F2711	14.62	31.06	18.44	35.88
F2715	14.24	37.02	21.27	27.47
F2719	27.29	57.24	14.14	1.33
F2720	9.90	48.32	28.69	13.09
F2843	2.01	44.30	42.45	11.24
F2842	19.63	42.62	34.06	3.69
F2847	16.81	52.25	30.95	0.00
F2848	15.08	43.72	19.60	21.61
F2850	13.07	56.28	16.58	14.07
F4512	7.81	53.65	11.63	26.91
F4513	7.54	56.62	24.46	11.39
F4527	27.79	28.79	8.99	34.44
F4528	17.61	68.44	8.97	4.98
F4535	27.85	51.68	8.73	11.75
F4536	15.91	55.44	21.44	7.20
F4567	15.41	27.47	37.69	19.43
F4568	20.13	51.91	17.97	9.98
F4587	14.95	31.06	17.77	36.21
F5714	14.93	46.31	27.35	11.41
F5715	8.88	42.71	27.97	20.44
F5733	26.17	58.89	9.06	5.87
F5734	17.45	30.37	21.81	30.37
F5740	25.58	64.29	0.17	9.97
F6302	5.16	20.63	19.30	54.91
F6308	3.32	76.91	18.27	1.50
F6310	21.30	15.97	10.65	52.08
F6324	14.98	24.79	18.64	41.60
F6325	36.94	48.42	0.50	14.14
F7503	1.00	25.46	42.26	31.28
F7513	24.29	24.96	23.63	27.12
F7520	0.00	53.49	39.54	6.98
F7523	20.76	58.47	10.47	10.30
**Average**	**15.89**	**44.67**	**20.42**	**19.02**
SE	**1.55**	**2.66**	**1.96**	**2.58**
SQRT(N)	5.66	5.66	5.66	5.66

PDAPP 10 min

Animal	Quadrant time (%) adj L	Training	adj R	Opp
F2728	15.58	28.14	30.49	25.80
F2713	10.05	51.26	28.31	10.39
F2717	14.48	61.23	20.80	3.49
F2718	32.50	57.79	7.20	2.51
F2845	21.64	65.77	11.75	0.84
F2851	21.63	50.08	19.30	8.99
F2852	7.89	62.92	27.35	1.85
F2853	47.15	34.23	8.73	9.90
F4516	15.14	51.91	26.96	5.99
F4517	18.44	63.62	13.95	3.99
F4531	25.96	48.07	12.23	13.74
F4533	19.14	26.46	20.47	33.94
F4534	17.25	21.27	36.85	24.62
F4566	28.07	34.88	12.96	24.09
F4588	20.10	33.33	29.48	17.09
F5717	12.79	23.75	36.21	27.24
F5741	12.25	42.95	26.68	18.12
F6323	21.61	46.06	8.38	23.95
F7514	27.69	20.13	14.93	37.25
F7515	15.12	53.99	19.27	11.63
F7521	6.15	45.35	39.54	8.97
F7522	19.10	64.99	13.40	2.51
F7532	19.10	58.46	9.05	13.40
**Average**	**19.51**	**45.51**	**20.62**	**14.36**
SE	**1.84**	**3.12**	**2.07**	**2.24**
SQRT(N)	4.80	4.80	4.80	4.80

PDAPP 7 days

Animal	Quadrant time (%) adj L	Training	adj R	Opp
F2728	22.32	28.86	32.55	16.28
F2713	8.31	36.38	45.18	10.13
F2717	18.97	42.93	13.98	24.13
F2718	48.59	31.61	5.16	14.64
F2845	0.00	37.25	55.87	6.88
F2851	5.98	13.46	35.55	45.02
F2852	2.69	59.06	33.05	5.20
F2853	33.73	32.22	17.11	16.95
F4516	14.31	39.43	36.27	9.98
F4517	8.31	30.40	38.21	23.09
F4531	32.05	47.99	9.90	10.07
F4533	21.48	25.00	23.83	29.70
F4534	22.92	25.91	25.58	25.58
F4566	22.80	22.96	26.96	27.29
F4588	40.03	21.43	26.25	12.29
F5717	15.58	25.96	32.83	25.63
F5741	19.77	14.95	14.95	50.33
F6323	29.70	35.57	18.12	16.61
F7514	19.97	26.79	20.80	32.45
F7515	20.47	45.59	20.97	12.98
F7521	15.94	44.63	35.24	4.20
F7522	31.95	53.41	9.65	4.99
F7532	32.83	23.45	14.07	29.65
**Average**	**21.25**	**33.27**	**25.74**	**19.74**
SE	**2.51**	**2.46**	**2.59**	**2.58**
SQRT(N)	4.80	4.80	4.80	4.80

Supplementary References

1. K. J. Hayes, The backward curve: a method for the study of learning. *Psychological Review* **60**, 269-275. (1953).
2. R. I. Spooner, A. Thomson, J. Hall, R. G. M. Morris, S. H. Salter, The Atlantis platform: a new design and further developments of Buresova's on-demand platform for the water maze. *Learning & memory* **1**, 203-211 (1994).
